# Prognostic Value for Mortality of Plasma Bioactive Adrenomedullin in Patients with Pulmonary Arterial Hypertension: A Sub Analysis of the Biomarker Study in the COHARD-PH Registry

**DOI:** 10.3390/medicina59040748

**Published:** 2023-04-12

**Authors:** Anggoro Budi Hartopo, Dyah Wulan Anggrahini, Lucia Kris Dinarti, Anne-Kathrin Schäfer, Andreas Bergmann, Jajah Fachiroh, Salvatore Di Somma

**Affiliations:** 1 Department of Cardiology and Vascular Medicine, Dr. Sardjito Hospital, Faculty of Medicine, Public Health and Nursing, Universitas Gadjah Mada, Yogyakarta 55281, Indonesia; 2 SphingoTec GmbH, Hennigsdorf, 16761 Berlin, Germany; 3 Department of Histology and Cell Biology, Faculty of Medicine, Public Health and Nursing, Universitas Gadjah Mada, Yogyakarta 55281, Indonesia; 4 Biobank Unit, Faculty of Medicine, Public Health and Nursing, Universitas Gadjah Mada, Yogyakarta 55281, Indonesia; 5 Department of Medical-Surgery Sciences and Translational Medicine, Faculty of Medicine and Psychology, Sapienza University of Rome, 00185 Rome, Italy; 6 GREAT Network, 00191 Rome, Italy

**Keywords:** pulmonary arterial hypertension, idiopathic/heritable pulmonary arterial hypertension, atrial septal defects-associated pulmonary arterial hypertension, bioactive adrenomedullin, prognosis, mortality

## Abstract

The adrenomedullin level increases in pulmonary arterial hypertension (PAH, and correlates with a high mortality rate. Its active form, bioactive adrenomedullin (bio-ADM), has been recently developed and has significant prognostic applications in acute clinical settings. Aside from idiopathic/hereditary PAH (I/H-PAH), atrial septal defects-associated pulmonary artery hypertension (ASD-PAH) is still prevalent in developing countries and associated with increased mortality. This study aimed to investigate the mortality-wise prognostic value of the plasma bio-ADM level by comparing subjects with ASD-PAH and I/H-PAH with ASD patients without pulmonary hypertension (PH) as a control group. This was a retrospective, observational cohort study. The subjects were Indonesian adult patients who were recruited from the Congenital Heart Disease and Pulmonary Hypertension (COHARD-PH) registry and divided into three groups: (1) ASD without PH (control group), (2) ASD-PAH and (3) I/H-PAH. During right-heart catheterization at the time of diagnosis, a plasma sample was taken and assayed for bio-ADM using a chemiluminescence immunoassay. Follow-up was performed as a part of the COHARD-PH registry protocol in order to evaluate the mortality rate. Among the 120 subjects enrolled: 20 turned out to have ASD without PH, 85 had ASD-PAH and 15 had I/H-PAH. Compared to the control group (5.15 (3.0–7.95 pg/mL)) and ASD-PAH group (7.30 (4.10–13.50 pg/mL)), bio-ADM levels were significantly higher in the I/H-PAH group (median (interquartile range (IQR)): 15.50 (7.50–24.10 pg/mL)). Moreover, plasma bio-ADM levels were significantly higher in subjects who died (n = 21, 17.5%) compared to those who survived (median (IQR): 11.70 (7.20–16.40 pg/mL) vs. 6.90 (4.10–10.20 pg/mL), *p* = 0.031). There was a tendency toward higher bio-ADM levels in those who died among the PAH subjects, in both ASD-PAH and I/H-PAH groups. In conclusion, the plasma bio-ADM level is elevated in subjects with PAH from both ASD-PAH and I/H-PAH origins, reaching the highest levels in subjects with the I/H-PAH form. A high bio-ADM level tended to be associated with a high mortality rate in all subjects with PAH, indicating a relevant prognostic value for this biomarker. In patients with I/H-PAH, monitoring bio-ADM could represent a valid tool for predicting outcomes, allowing more appropriate therapeutical choices.

## 1. Introduction

Adrenomedullin is a peptide present in human tissues and highly expressed in vasculature [1]. It is synthesized as a preprohormone and processed to proadrenomedullin, which is then fragmented into four segments, namely, proadrenomedullin N-terminal 20 peptide (PAMP)-Gly, mid-regional pro-adrenomedullin (MR-proADM), adrenomedullin-Gly, and C-terminal proadrenomedullin [1]. Both PAMP and adrenomedullin are processed as intermediate forms, which are biologically inactive, and the peptides are then converted to their mature bioactive forms [1]. The mature bioactive adrenomedullin has a short half-life in circulation and non-specific binding to surfaces, which makes it difficult to reliably measure [2]. Currently, a new assay with which to reliably measure mature bioactive adrenomedullin or bio-ADM has been developed, and the results showed significant applications in acute clinical settings [3,4,5,6]. 

Pulmonary arterial hypertension (PAH) is a progressive and debilitating disease, distinguished by occlusive remodeling in the distal pulmonary vasculature [7]. The current mortality rate among patients with PAH is still high, reaching 20% in a 3-year follow-up [7]. In those with high-risk stratification at the time of diagnosis, the 3-year mortality rate is up to 55% [7]. This devastating outcome prompts the efforts to elucidate potential biomarkers for better risk stratification and eventually modify treatment strategies.

Plasma MR-proADM with adrenomedullin precursors, and peptide levels have been shown to be increased in patients with PAH and linked with a high mortality rate [8,9]. Inhalation of adrenomedullin significantly reduced mean pulmonary artery pressure (mPAP) and pulmonary vascular resistance (PVR) among patients with idiopathic PAH [10]. These findings indicate that adrenomedullin could play a functional role in the pathophysiology of PAH with several different aspects [10]. Current clinical classification divided PAH into idiopathic/heritable PAH (I/H-PAH) and PAH due to associated conditions [11]. In developed countries, I/H PAH is the most common form, whereas in developing countries, congenital heart disease-associated PAH was the most dominant form [11]. Our registry showed that, among congenital heart diseases, uncorrected atrial septal defects-associated pulmonary artery hypertension (ASD-PAH) is the most prevalent [12,13]. This study was designed to investigate the prognostic value regarding mortality of plasma bio-ADM levels in subjects with ASD-PAH or idiopathic/hereditary PAH (I/H PAH).

## 2. Materials and Methods

### 2.1. Subjects’ Enrollment

This was a retrospective, observational cohort study. The subjects were Indonesian adult patients (>18 years old) who were recruited from the Congenital Heart Disease and Pulmonary Hypertension (COHARD-PH) registry. The COHARD-PH registry is a single center, hospital-based registry which enrolls adult patients with congenital heart diseases with/without PAH and other forms of PAH in Dr. Sardjito Hospital, a PH-dedicated center in Yogyakarta, Indonesia [13]. We selected subjects to be included in this study, namely: patients with (1) ASD without PH (control group), (2) ASD-PAH and (3) I/H-PAH. Group (1): patients with large secundum ASD (≥2 cm diameter of ASD) and mean pulmonary artery pressure (mPAP) ≤ 20 mmHg. Group (2): patients with large secundum ASD, mPAP > 20 mmHg, pulmonary vascular resistance (PVR) > 2 Wood units and pulmonary artery wedge pressure (PAWP) or left atrial pressure (LAP) ≤ 15 mmHg. Group (3): patients with mPAP > 20 mmHg, PVR > 2 Wood units and PAWP ≤ 15 mmHg and without any secondary cause of PAH. The hemodynamic measurements (mPAP, PVR, PVR index, PAWP and LAP) were performed by right-heart catheterization (RHC) using indirect Fick methods [13]. The inclusion criteria were patients from the COHARD-PH registry who had: (1) ample echocardiography data, (2) complete hemodynamic data from RHC, (3) sufficient diagnosis procedure for I/H-PAH and (4) blood samples stored in the Biobank Unit. We excluded patients who: (1) underwent ASD closure during the follow-up period, (2) did not have follow-up data in the COHARD-PH database and (3) did not have sufficient blood samples in the Biobank Unit, Faculty of Medicine, Public Health or Nursing Universitas Gadjah Mada, Yogyakarta, Indonesia. The RHC was performed in stable patients; therefore, the systemic complications, such as sepsis, left heart failure and shocks, were null. 

All subjects underwent transthoracic echocardiography, transesophageal echocardiography and RHC, at the time of diagnosis, based on the COHARD-PH enrollment criteria [13]. The diagnosis of PAH in subjects with ASD-PAH or I/H PAH was based on hemodynamic measurements by RHC according to current international criteria of PAH [11,14]. A number of diagnostic procedures, such as thorax high resolution computerized tomography (HRCT) scanning, cardiac multislice computerized tomography (MSCT) scanning, CT pulmonary angiography, cardiac magnetic resonance imagery (MRI) and spirometry, were performed in selected patients to guide the diagnosis of I/H-PAH [13]. The baseline data collection, including plasma biomarkers, was conducted before PAH-specific treatments were given to the patients.

All subjects signed an informed consent form to participate in the study. The Medical and Health Research Ethic Committee Faculty of Medicine, Public Health and Nursing Universitas Gadjah Mada-Dr. Sardjito Hospital approved the study protocol (Ref. number: KE/FK/1189/EC/2021).

### 2.2. Biomarker Procedures

During RHC, the fasting blood samples taken from inferior vena cava were collected in EDTA-tubes, centrifuged at 3500× *g* for 15 min, aliquoted and stored in −80 °C in the Biobank Unit, Faculty of Medicine, Public Health and Nursing, Universitas Gadjah Mada, Indonesia, until assayed for bio-ADM. An aliquoted sample was thawed, and bio-ADM was measured using a chemiluminescence immunoassay (sphingotest^®^ bio-ADM^®^, SphingoTec GmbH, Hennigsdorf, Germany) as previously described [6].

The hemoglobin concentration, hematocrit percentage and N terminal-pro brain natriuretic peptide (NT-proBNP) level (electrochemiluminescence immunoassay (ElecsysProBNP II) and a Cobas e immunoassay analyzer (Roche Diagnostics, Germany)) were measured using blood samples taken during RHC at index of diagnosis in Dr. Sardjito Hospital central laboratory, as previously described [13]. 

### 2.3. Mortality Outcomes

The subject follow-up was performed as a part of the COHARD-PH registry protocol. Subjects were visited monthly at the Dr. Sardjito PH outpatient clinic for regular monitoring. Those who did not come to the PH outpatient clinic were followed-up by phone or online messaging services. The mortality outcomes during the follow-up period were kept in the COHARD-PH database. We retrieved and analyzed the subjects’ mortality data from the registry. The follow-up period for patients’ new visits ranged from a minimum of 6 months up to 8 years. 

### 2.4. Statistical Analysis

The numerical data were tested for normality distribution by the Kolmogorov–Smirnov test and are presented as means and standard deviations (SDs) for normally distributed numerical data or median and interquartile range (IQR) for not normally distributed numerical data. The categorical data are presented as sums and percentages. The comparison of numerical data between two groups was performed with Student’s T or Mann–Whitney tests where applicable, and one-way ANOVA tests were used for more than two groups. The categorical data were compared with chi-square tests. A *p* value < 0.05 was considered statistically significant.

## 3. Results

### 3.1. Bio-ADM 

We selected 120 subjects: 20 patients with ASD without PH (control group), 85 patients with ASD-PAH and 15 patients with I/H-PAH. Table 1 shows the subjects characteristics. 

**Figure 1:**
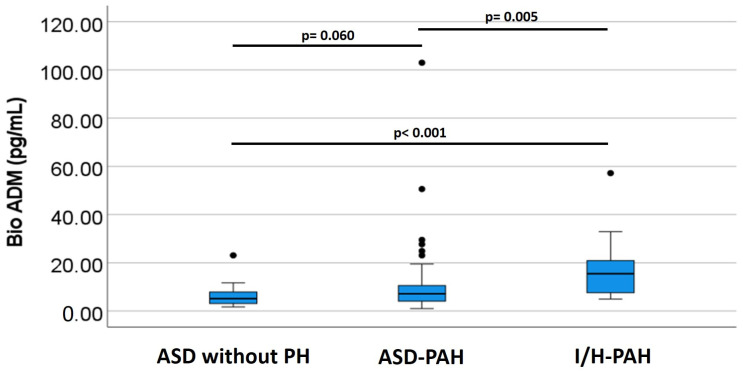
The bio-ADM levels of the groups. The highest bio-ADM level was observed in subjects with I/H-PAH, followed by those with ASD-PAH. ASD without PH was the control group. The Mann–Whitney test: ASD without PH vs. ASD-PAH, *p* = 0.060; ASD without PH vs. I/H-PAH, *p* < 0.001; ASD-PAH vs. I/H-PAH, *p* = 0.005.

The bio-ADM levels were significantly higher in subjects with I/H-PAH (median (IQR): 15.50 (7.50–24.10) pg/mL) compared to those with ASD-PAH (median (IQR): 7.30 (4.10–13.50) pg/mL) and with ASD without PH (median (IQR): 5.15 (3.0–7.95) pg/mL), *p* = 0.024. The levels of bio-ADM in the groups and the analysis are shown in Table 2 and Figure 1. 

### 3.2. Bio-ADM and Mortality 

In all subjects, twenty-one (17.5%) died during the follow-up period. The comparison of subjects’ characteristics between those who died (mortality (+)) and those who survived (mortality (−)) is shown in Table 3. All subjects with ASD without PH (control group) survived. Of those who died, 61.9% were in the ASD-PAH group and 38.1% were subjects in the I/H PAH group. 

The plasma level of bio-ADM was significantly higher in all subjects who died compared to those who survived (median (IQR): 11.70 (7.20–16.40) pg/mL vs. 6.90 (4.10–10.20) pg/mL, *p* = 0.031). Among subjects with ASD-PAH, there was a tendency toward higher plasma bio-ADM in those who died than in those who survived (median (IQR): 10.00 (4.45–13.55) pg/mL vs. 7.15 (4.10–13.45) pg/mL, *p* = 0.580). In subjects with I/H-PAH, the plasma bio-ADM level also tended to be higher in subjects who died compared to those who survived (median (IQR): 16.45 (8.90–24.23) pg/mL vs. 9.80 (6.50–24.10) pg/mL, *p* = 0.487). Table 4 shows the plasma levels of bio-ADM of subjects based on mortality status.

## 4. Discussion

The main findings of this study were that: (1) plasma bio-ADM level was elevated in patients with I/H-PAH, compared with ASD patients without PH (control group) and the ASD-PAH group; (2) the plasma bio-ADM level was tended to be higher in patients with ASD-PAH as compared to controls; and (3) a higher plasma bio-ADM level tended to be associated with a higher mortality rate in subjects with PAH, both ASD-PAH and I/H-PAH ones. 

Currently, four PAH-related molecular pathways have been fruitfully exploited as treatment modalities, namely, voltage-gated, L-type calcium channels and nitric oxide cyclic guanosine monophosphate, endothelin and prostacyclin pathways, resulting in fourteen drugs which improve the quality of life and lengthen the time till clinical worsening, but not mortality [15]. The current classes of PAH-specific drugs primarily target vasoconstriction. However, other main mechanisms of PAH pathogenesis which drive pathological pulmonary vascular remodeling have not been explored, such as inflammation, thrombosis, hyperproliferative and apoptosis-resistant pulmonary artery smooth muscle cells, and disproportionate fibrosis [15]. The potential of adrenomedullin and bio-ADM as functional biomarkers to become targeted PAH treatment has been proposed considering its impacts on inflammation, vasoconstriction and thrombosis [15]. 

Adrenomedullin is expressed in endothelia and vascular smooth muscle cells, which functions to vasodilate and maintain vascular integrity through direct action on vascular smooth muscle cells and the formation of nitric oxide [16]. In a therapeutic approach, adrenomedullin administration has been shown to reduce blood pressure in the vessels but increase the blood flow [17]. Moreover, the infusion of adrenomedullin into patients with PH reduced PVR and mPAP and decreased plasma aldosterone and BNP levels [18]. This suggests a positive relationship between ADM-induced vasodilatation and better outcomes in PAH patients. For the diagnostic approach, the baseline adrenomedullin level was associated with a higher risk score for PAH prognosis, as levels of MR-proADM along with plasma adrenomedullin peptides and precursors are elevated in patients with PAH compared to healthy subjects [8,9]. Here, the increased adrenomedullin levels were associated with worsened markers of PAH severity—namely, a higher NT-proBNP level, higher mean right atrial pressure (mRAP), higher PVR, a shorter 6 min walking distance and a lower cardiac index [8,9]. Even though we used different backgrounds of PAH, namely, ASD-PAH and ASD without PH as controls, and the type of adrenomedullin measured in our study differs from those previously reported, since we measured the bioactive adrenomedullin or bio-ADM, the results from previously mentioned studies could still be confirmed. 

In our study, we obtained new evidence that in adult patients with ASD, an elevated plasma bio-ADM level may indicate the ongoing presence of PAH. In large ASD, a left-to-right shunt facilitates a significant increase in pulmonary blood flow overtime. This volume overflow induces various pathophysiology mechanisms in pulmonary vasculature—namely: elevated shear stress, endothelial dysfunction, vascular smooth muscle hypertrophy and neointimal proliferation; and finally, alteration of the pulmonary vasculature or remodeling, all of which contribute to the progress toward PAH [19]. Our selected subjects were patients with uncorrected ASD, and we showed that in those who had already developed PAH, the plasma level of bio-ADM tended to be higher. Moreover, among subjects with ASD-PAH, there was a tendency toward a higher plasma bio-ADM level in those who died than in those who survived. 

To establish bio-ADM as a prognostic marker in patients with PAH associated with ASD, or with congenital heart diseases (CHD) in general, measurements need to be rolled out more broadly in larger numbers of subjects and over longer follow-up periods. An elevated plasma bio-ADM level in ASD-PAH is aligned with an augmented NT-proBNP level, an established biomarker for detection of PAH development to indicate ASD and PAH severity [11,20]. In our study, the NT-proBNP level was higher in ASD-PAH as compared to I/H-PAH, which was the opposite to the plasma bio-ADM level. Since the hemodynamic of ASD-PAH was the combination of overtime volume and pressure overloads impacted whole cardiac chambers, the NT-proBNP level was exceedingly increased as compared to other forms of PAH [20]. Furthermore, the impact of PAH-specific treatment on plasma bio-ADM level needs to be investigated. Previous study showed that plasma adrenomedullin peptides and precursor levels did not change after PAH-specific treatment [9]; however, in the patients with CHD-associated PAH, these data have not been evaluated. 

Adrenomedullin-specific receptor (AM1) is abundantly expressed on the endothelia of alveolar capillaries in healthy individuals [21]. Its expression is constitutively maintained by the basal adrenomedullin level, and it acts as a clearance receptor for adrenomedullin [22]. Using molecular imaging techniques, the heterogeneity index of AM1 is higher in pulmonary vasculature among subjects with PAH compared to healthy controls [23]. The net reduction of AM1 receptors in the lungs may explain the elevated plasma level of bio-ADM, since its clearance receptors are diminished. Furthermore, the elevated plasma bio-ADM level in patients with PAH reflects the altered vascular regulation and the severity of pulmonary vascular remodeling in both ASD-PAH and I/H-PAH. Our previous study in subjects with ASD-associated PAH indicated that the potent vasoconstrictor endothelin-1 was associated with the severity of PAH, whereas vasodilators, prostacyclin and nitric oxide were not differential [24]. In this current study, plasma bio-ADM could be a marker of pulmonary vascular remodeling, which leads to increased PVR and finally PAH, in subjects with ASD. Previous study in sepsis patients showed that a higher level of bio-ADM, >70 pg/mL on admission and persisting for 4 days, was associated with a higher probability of mortality in 28 days [3]. Furthermore, in acute-heart-failure patients, a high bio-ADM level, ≥43 pg/mL, was associated with an increase in 1-year mortality [6]. As a side note, the bio-ADM measurements in both previous studies were conducted in acute settings, whereas in this study, stable chronic PAH patients were analyzed, which could explain the lower value of bio-ADM we obtained.

In patients with sepsis and cardiogenic shocks, recent studies showed the beneficial impact of adrecizumab, a humanized antibody directed against the N-terminus of adrenomedullin [25,26,27]. Adrecizumab promotes protective effects mediated by adrenomedullin on the endothelial intact barrier, by binding to circulating adrenomedullin and attracting interstitial adrenomedullin into circulation [28]. Considering the pulmonary vascular resistance improvement after administration of adrenomedullin infusion [18], adrecizumab may have a potential therapeutic application in patients with PAH, both ASD-PAH and I/H-PAH patients. Further research is needed to support and prove this hypothesis.

### The Study’s Limitations

This study has some limitations that could be summarized as:

(1) A rather small number of subjects with I/H PAH were enrolled, leading to the need to confirm these data in a new multicentric prospective study with more patients from these groups.
(2) The biomarkers’ results were obtained analyzing blood samples that were stored and frozen for a long period, and we cannot exclude the possibility that this could have affected the results. Consequently, it will be necessary to confirm the results of biomarker data obtained from this study and analyze fresh plasma samples in the future.
(3) The use of medication with PAH-specific drugs was quite limited in our COHARD-PH registry, since not all PAH-specific drugs are available in Indonesia.

## 5. Conclusions

In conclusion, the plasma bio-ADM level is increased in subjects with I/H-PAH, as compared to ASD without PH and ASD-PAH. Compared to ASD without PH, the plasma bio-ADM level tends to be higher in patients with ASD-PAH. During follow-up, a higher bio-ADM level tends to be associated with a high mortality rate in all subjects, indicating prognostic value for this biomarker. From our study, regular, timely monitoring of these patients with bio-ADM seems to be useful for further therapeutic decision making. Further multicenter studies are needed to corroborate this finding. 

## Figures and Tables

**Table 1:** Characteristics of subjects—patients with ASD without PH as the control group, ASD-PAH patients and I/H PAH patients.

Parameters	Control Group (n = 20)	ASD-PAH (n = 85)	I/H PAH (n = 15)	*p* Value
Age, years (mean ± SD)	30.70 ± 7.63	36.45 ± 11.60	39.33 ± 10.80	0.049
Sex				
Female, n(%) (n = 99 (82.5%)	18 (90.0)	69 (81.2)	12 (80.0)	0.623
Male, n(%) (n = 21 (17.5%)	2 (10.0)	16 (18.8)	3 (20.0)	
LV EF, % (mean ± SD)	69.70 ± 6.63	71.52 ± 9.07	NA	0.313
TAPSE, mm (mean ± SD)	27.80 ± 3.65	23.59 ± 5.89	17.50 ± 7.05 (n = 12)	<0.001
TV gradient, mmHg (mean ± SD)	28.11 ± 9.40	84.90 ± 31.09	85.00 ± 18.38 (n = 2)	<0.001
Hemoglobin, g/dL (mean ± SD)	12.84 ± 1.21	14.49 ± 2.30	13.93 ± 1.81	0.008
Hematocrit, % (mean ± SD)	38.93 ± 3.03	43.68 ± 6.37	42.40 ± 4.77	0.005
NT-proBNP, pg/mL (mean ± SD)	107.45 ± 95.39 (n = 13)	2256.32 ± 3497.64 (n = 69)	1566.36 ± 1143.37 (n = 14)	0.063
mPAP, mmHg (mean ± SD)	17.40 ± 2.21	56.93 ± 17.57	64.27 ± 17.13	<0.001
PVR index, WU/m^2^ (mean ± SD)	1.46 ± 0.75	16.31 ± 16.55	31.32 ± 10.89	<0.001
mRAP, mmHg (mean ± SD)	5.72 ± 3.34 (n = 18)	11.83 ± 6.28	13.73 ± 7.74	<0.001
SaO_2_ Aorta, % (mean ± SD)	96.23 ± 1.72	88.97 ± 6.32 (n = 80)	90.35 ± 6.39	<0.001

ASD: atrial septal defect; PH: pulmonary hypertension; PAH: pulmonary artery hypertension; I/H: idiopathic/hereditary; LV EF: left ventricle ejection fraction; TAPSE: tricuspid annular plane systolic excursion; TV: tricuspid valve; NT-proBNP: N terminal-pro brain natriuretic peptide; mPAP: mean pulmonary artery pressure; PVR: pulmonary vascular resistance; WU: Wood unit; mRAP: mean right atrial pressure; SaO_2_: oxygen saturation; SD, standard deviation.

**Table 2:** The bio-ADM (pg/mL) levels among subjects with ASD without PH (control group), ASD-PAH and I/H- PAH.

Bio-ADM, pg/mL	Control Group (n = 20)	ASD-PAH (n = 85)	I/H PAH (n = 15)
Mean ± SD *	6.33 ± 4.79	10.55 ± 12.82	17.65 ± 13.62
Median (IQR)	5.15 (3.0–7.95)	7.30 (4.10–13.50)	15.50 (7.50–24.10)

ASD, atrial septal defect; Bio-ADM, bioactive adrenomedullin; IQR, interquartile range; SD, standard deviation; * one-way ANOVA test: *p* = 0.024.

**Table 3:** Comparison of characteristics between all subjects who died (mortality (+)) and those who survived (mortality (−)).

Parameters	Mortality (+) (n = 21)	Mortality (−) (n = 99)	*p* Value
Age, years (mean ± SD)	34.43 ± 11.49	36.15 ± 11.13	0.261
Sex			
Female, n (%)	21 (100)	78 (78.8)	0.023
Male, n (%)	0 (0)	21 (21.2)	
LV ejection fraction, % (mean ± SD)	68.08 ± 9.16 (n = 13)	71.61 ± 8.54 (n = 91)	0.085
TAPSE, mm (mean ± SD)	21.10 ± 6.65 (n = 20)	24.23 ± 6.05 (n = 96)	0.020
TV gradient, mmHg (mean ± SD)	76.58 ± 29.37 (n = 12)	74.26 ± 36.58 (n = 92)	0.417
Hemoglobin, g/dL (mean ± SD)	13.18 ± 1.49 (n = 20)	14.34 ± 2.26	0.014
Hematocrit, % (mean ± SD)	40.51 ± 4.14 (n = 20)	43.18 ± 6.23	0.035
NT-proBNP, pg/mL (mean ± SD)	2180.45 ± 3148.9 (n = 18)	1791.84 ± 3079.41 (n = 78)	0.316
mPAP, mmHg (median, IQR)	56.00 (45.00–67.00)	50.50 (34.75–66.00)	0.257 *
PVR index, WU/m^2^ (median, IQR)	15.30 (5.30–24.40) (n = 19)	7.93 (2.76–25.08) (n = 98)	0.185 *
mRAP, mmHg (mean ± SD)	13.52 ± 7.81	10.61 ± 6.16 (n = 96)	0.032
SaO2 Aorta, % (mean ± SD)	90.48 ± 6.10 (n = 19)	90.32 ± 6.48 (n = 94)	0.460
PAH, n (%)	21 (100.0)	79 (79.8)	0.022
Diagnosis			
ASD without PH	0 (0)	20 (20.2)	<0.001
ASD-PAH	13 (61.9)	72 (72.7)	
I/H-PAH	8 (38.1)	7 (7.1)	

ASD: atrial septal defect; PH: pulmonary hypertension; PAH: pulmonary artery hypertension; I/H: idiopathic/hereditary; LV: left ventricle; TAPSE: tricuspid annular plane systolic excursion; TV: tricuspid valve; NT-proBNP: N terminal-pro brain natriuretic peptide; mPAP: mean pulmonary artery pressure; PVR: pulmonary vascular resistance; WU: Wood unit; mRAP: mean right atrial pressure; SaO_2_: oxygen saturation; SD: standard deviation; IQR: interquartile range. * Non-parametric Mann–Whitney test.

**Table 4:** The plasma levels of bio-ADM in association with mortality in all subjects, ASD-PAH subjects and I/H-PAH subjects.

**All Subjects (n = 120)**	**Mortality (+)** **(n = 21)**	**Mortality (−)** **(n = 99)**	***p*** Value *
Mean ± SD, pg/mL	12.63 ± 8.11	10.33 ± 12.99	0.031
Median (IQR), pg/mL	11.70 (7.20–16.40)	6.90 (4.10–10.20)	
**ASD-PAH (n = 85)**	**Mortality (+)** **(n = 13)**	**Mortality (−)** **(n = 72)**	
Mean ± SD, pg/mL	10.69 ± 13.69	9.81 ± 6.45	0.580
Median (IQR), pg/mL	10.00 (4.45–13.55)	7.15 (4.10–13.45)	
**I/H-PAH (n = 15)**	**Mortality (+)** **(n = 8)**	**Mortality (−)** **(n = 7)**	
Mean ± SD, pg/mL	17.21 ± 3.12	18.14 ± 18.48	0.487
Median (IQR), pg/mL	16.45 (8.90–24.23)	9.80 (6.50–24.10)	

ASD: atrial septal defect; PAH: pulmonary artery hypertension; I/H: idiopathic/hereditary; SD: standard deviation; IQR: interquartile range. * Non-parametric Mann–Whitney test.

## Data Availability

Not applicable.
